# Supplementary material for: Simulating the Dynamic Intra-Tumor Heterogeneity and Therapeutic Responses
Source: Cancers (Basel). 2022 Mar 24;14(7):1645. doi: 10.3390/cancers14071645 (PMC8996855; doi:10.3390/cancers14071645)
Supplement: Supplementary file 1 [file cancers-14-01645-s001.zip › cancers-1623654-supplementary.pdf]

cancers

MDPI

Supplementary Materials: Simulating the Dynamic Intra-Tumor Heterogeneity and Therapeutic Responses

Yongjing Liu, Cong Feng, Yincong Zhou, Xiaotian Shao and Ming Chen

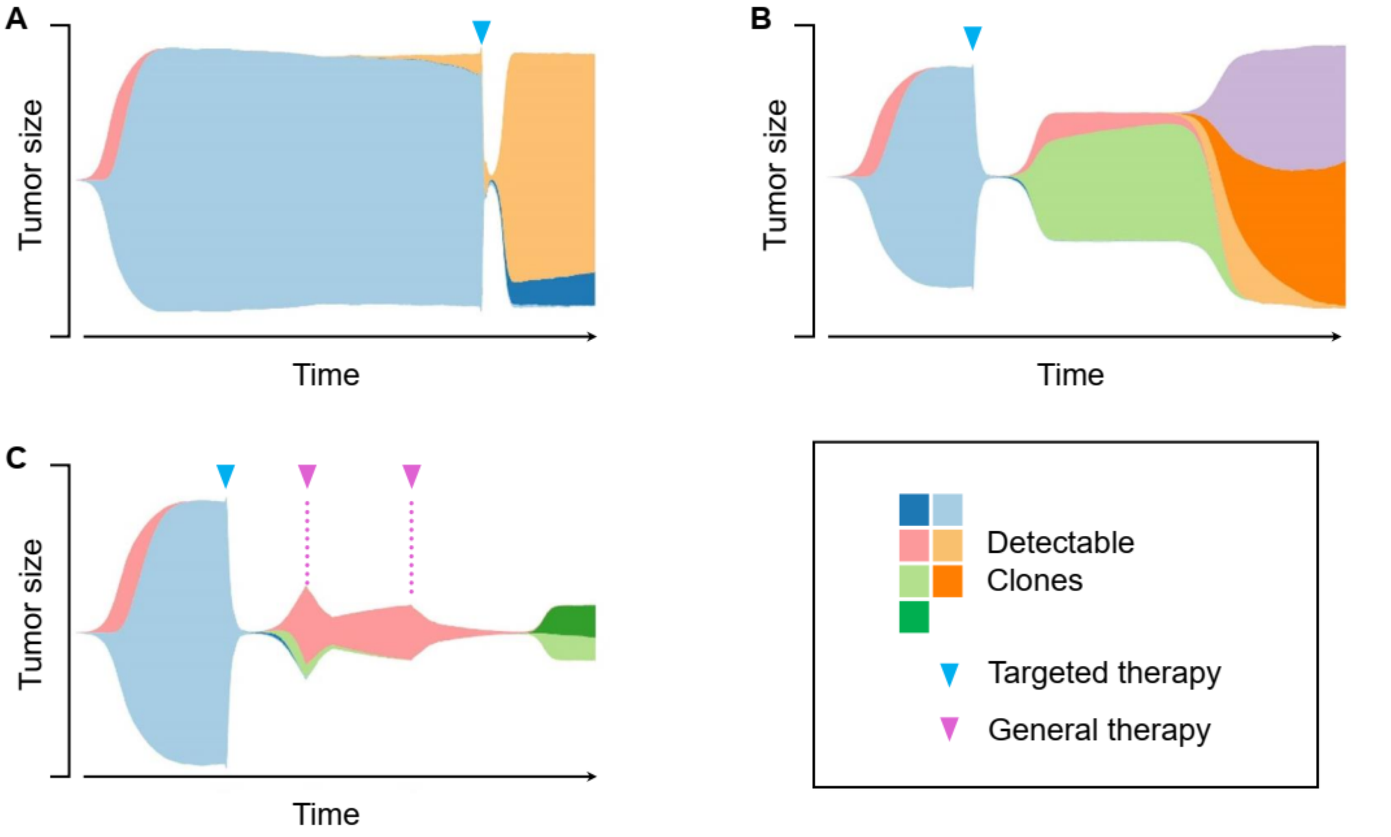

Figure S1. An illustration of tumor progression dynamics with therapeutic intervention. This figure shows examples of different therapeutic responses on the basis of Figure 2C. The blue triangle represents for a targeted therapy, the pink triangle represents for a general therapy. All these therapies are lasted for 10 iterations. Their position along the x axis indicates the time point of these therapies. (**A**) Rapid relapse after targeted therapy. (**B**) Target therapy when tumor heterogeneity is low. (**C**) Targeted therapy and subsequent adjuvant therapies.

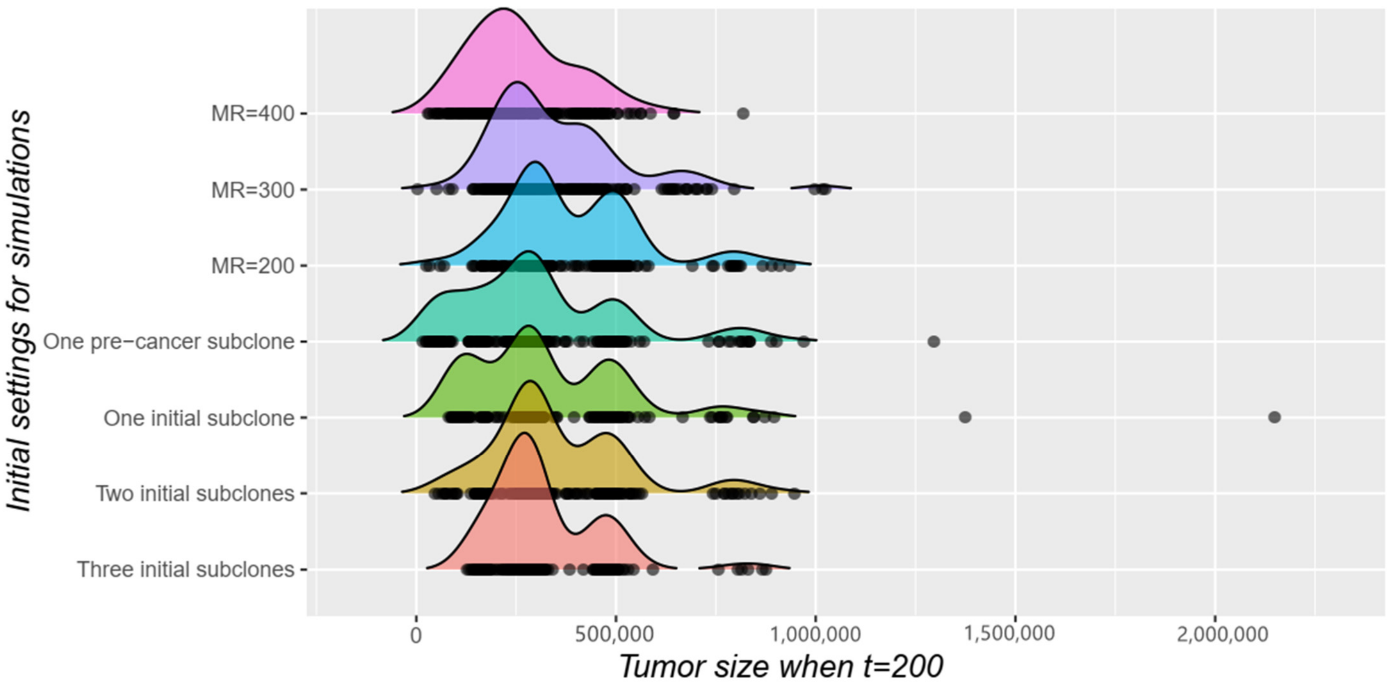

Figure S2. Distribution of the grown tumor sizes with different initial parameter settings. Ridge plot of the final tumor sizes with different initial conditions. The height of the plot indicates the local density. The x axis denotes the tumor size, the y axis denotes the initial condition. The simulations with each initial condition are repeated for 200 times, each represented by a point along the horizontal axes. For each simulation, 200 iterations are performed to obtain the final tumor size.

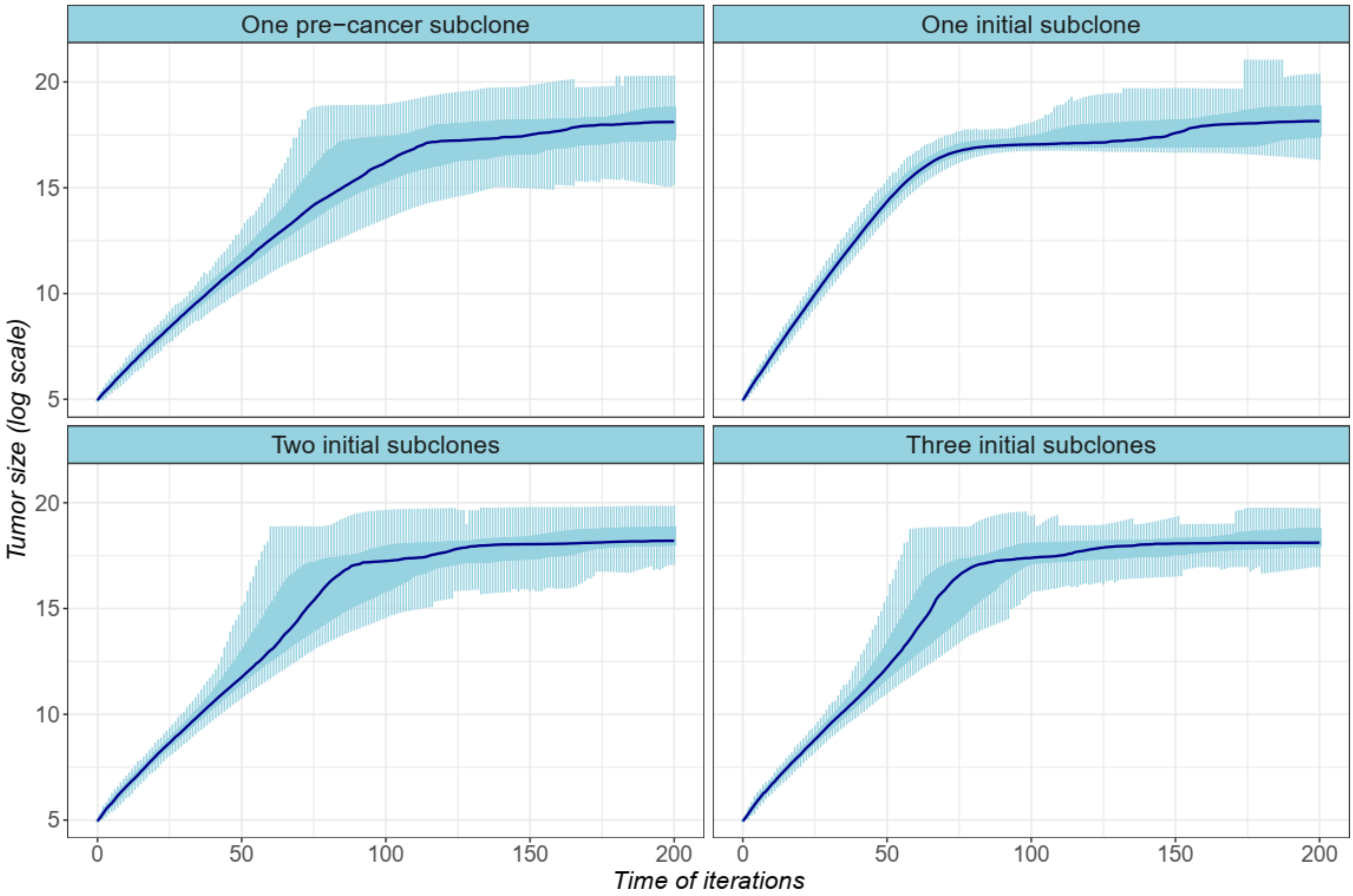

Figure S3. The dynamic progression of tumors with different rates of proliferation and numbers of initial subpopulations. Each panel represents a different setting of initial parameters, indicated by their titles. Boxplots are used to represent the distributions of tumor sizes from all the 200 simulations. The x axis denotes the time, and the y axis denotes the tumor size.

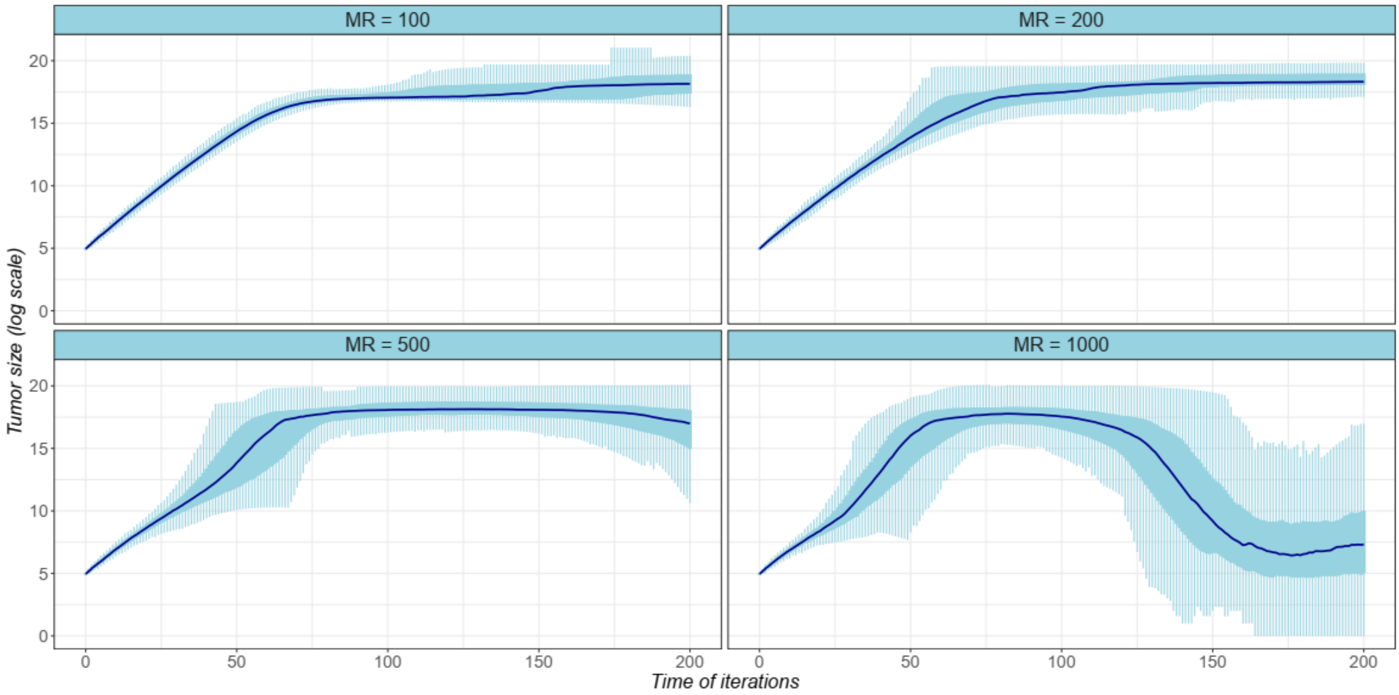

Figure S4. The dynamic progression of tumors with different initial mutation rates. Each panel represents a different setting of initial parameters, indicated by their titles. Boxplots are used to represent the distributions of tumor sizes from all the 200 simulations. The x axis denotes the time, and the y axis denotes the log-scaled tumor size.

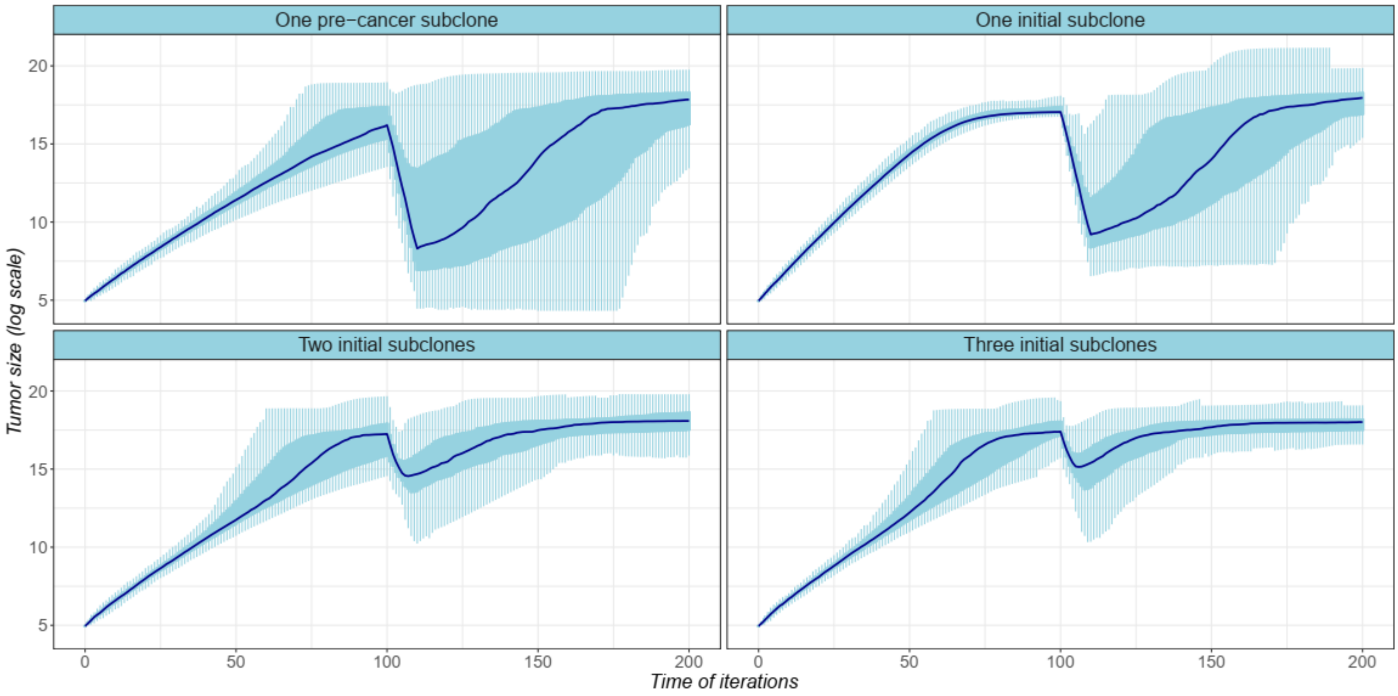

Figure S5. Simulations of the dynamic tumor progression after targeted therapy. Each panel represents a different setting of initial parameters, indicated by their titles. Boxplots are used to represent the distributions of tumor sizes from all the 200 simulations. The x axis denotes the time, and the y axis denotes the log-scaled tumor size.

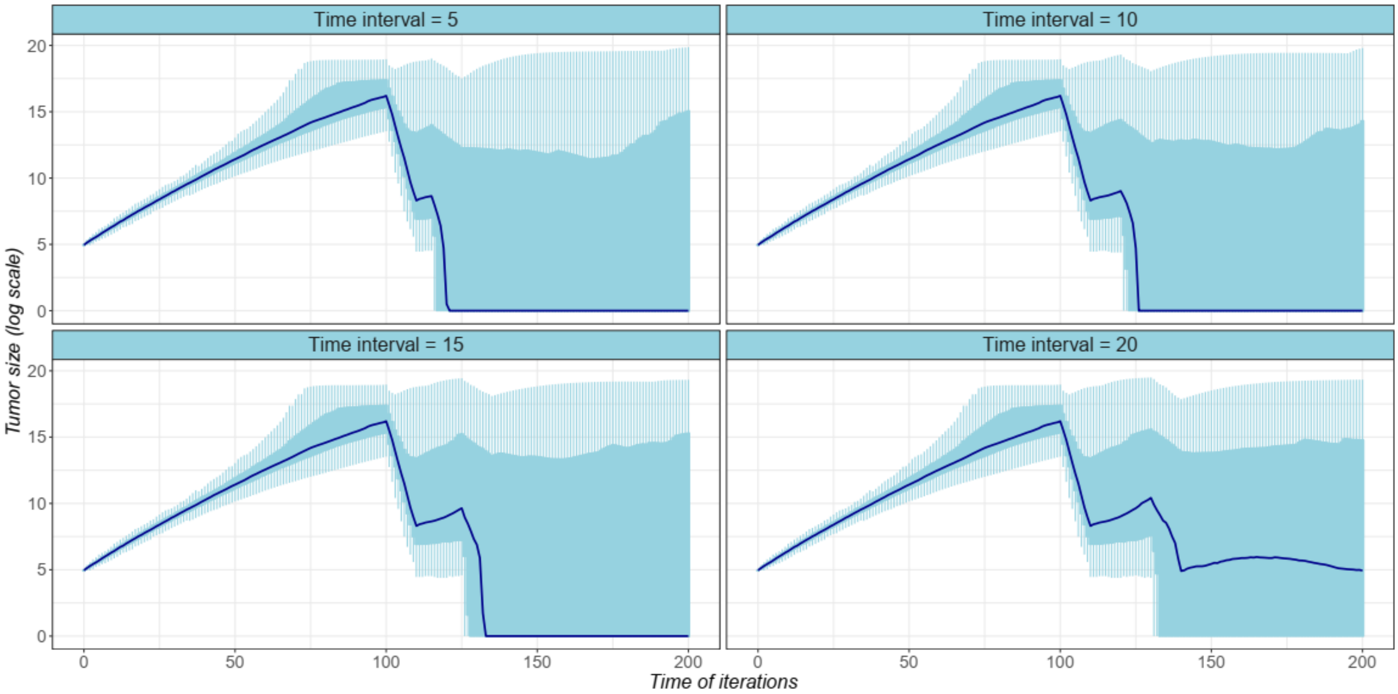

Figure S6. Simulations of the dynamic tumor progression after targeted therapy and an adjuvant general therapy. Each panel represents a different setting of initial parameters, indicated by their titles. Boxplots are used to represent the distributions of tumor sizes from all the 200 simulations. The x axis denotes the time, and the y axis denotes the log-scaled tumor size.

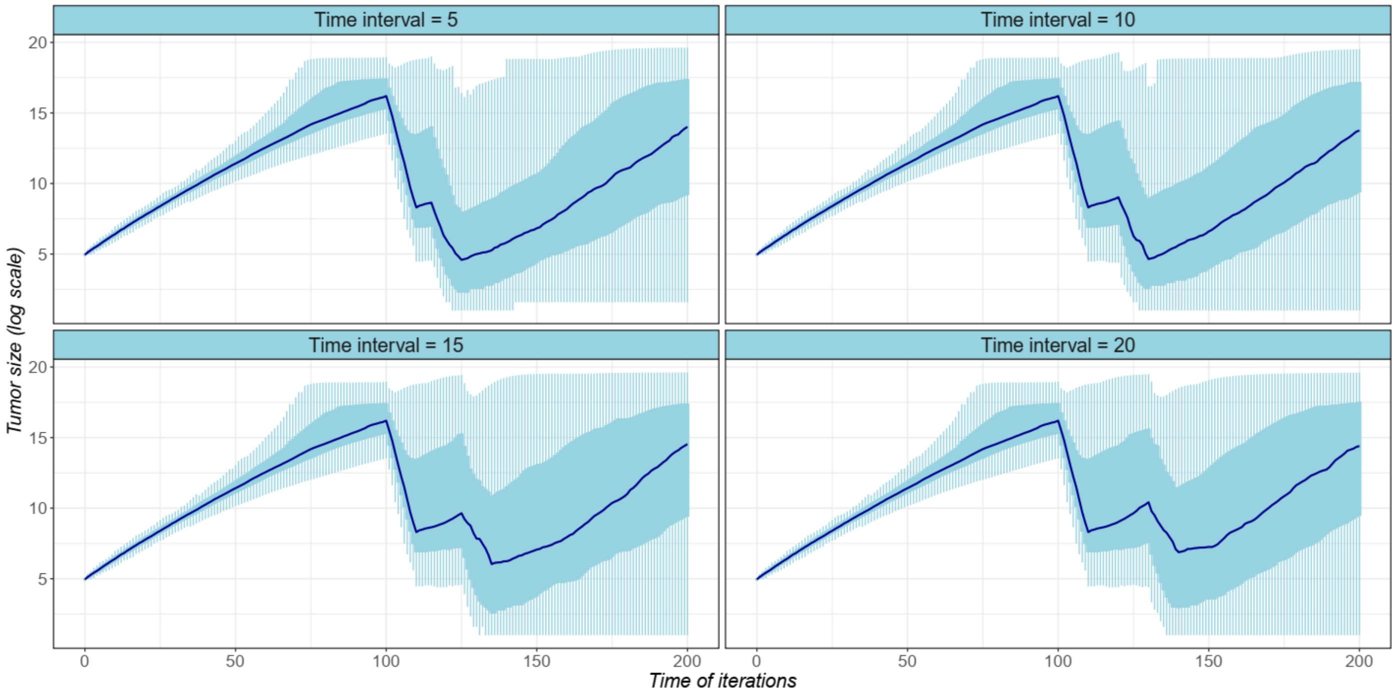

Figure S7. Simulations of the dynamic tumor progression after targeted therapy and an adjuvant targeted therapy. Each panel represents a different setting of initial parameters, indicated by their titles. Boxplots are used to represent the distributions of tumor sizes from all the 200 simulations. The x axis denotes the time, and the y axis denotes the log-scaled tumor size.

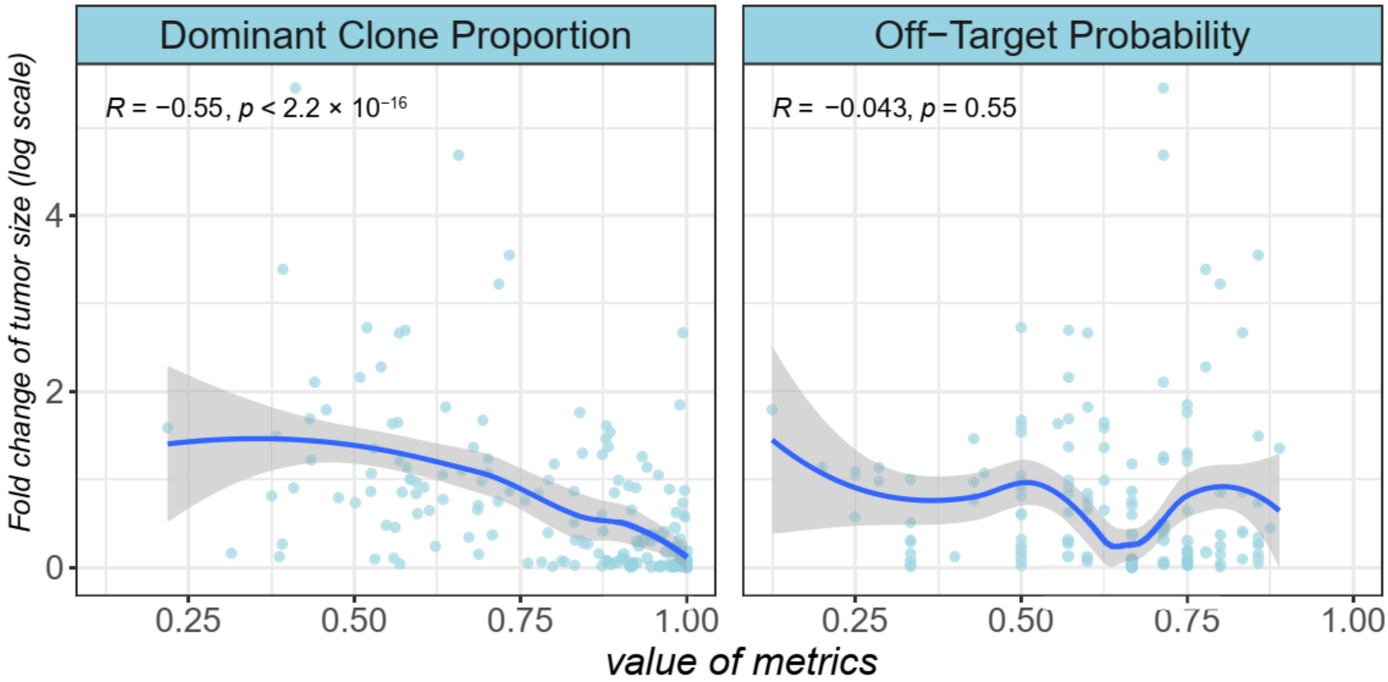

Figure S8. Correlations between the treatment outcome and the ITH metrics when all cells are originated from the first driver-carrying clone. Boxplots are used to represent the distributions of corresponding values from all the simulations. The x axis denotes the time, and the y axis denotes the value. 200 simulations are performed and two metrics are calculated at each time point. The dark blue line indicates the median value from all simulations, and the blue curve indicates a loess regression based on the values.
